# Exosome–transmitted long non-coding RNA *PTENP1* suppresses bladder cancer progression

**DOI:** 10.1186/s12943-018-0880-3

**Published:** 2018-10-03

**Authors:** Rui Zheng, Mulong Du, Xiaowei Wang, Weidong Xu, Jiayuan Liang, Wenying Wang, Qiang Lv, Chao Qin, Haiyan Chu, Meilin Wang, Lin Yuan, Jing Qian, Zhengdong Zhang

**Affiliations:** 1 Department of Environmental Genomics, School of Public Health, Jiangsu Key Laboratory of Cancer Biomarkers, Prevention and Treatment, Collaborative Innovation Center for Cancer Personalized Medicine, Nanjing Medical University, 101 Longmian Avenue, Jiangning District, Nanjing, 211166 China; 2 Department of Genetic Toxicology, The Key Laboratory of Modern Toxicology of Ministry of Education, School of Public Health, Nanjing Medical University, Nanjing, China; 3 Department of Biostatistics, School of Public Health, Nanjing Medical University, Nanjing, China; 4 Department of Urology, Yizheng Hospital, Drum Tower Hospital Group of Nanjing, Yizheng, People’s Republic of China; 5 Department of Urology, Beijing Friendship Hospital affiliated to Capital Medical University, Beijing, People’s Republic of China; 6 Department of Urology, The First Affiliated Hospital of Nanjing Medical University, Nanjing, China; 7 Department of Urology, Jiangsu Province Hospital of TCM, 155 Hanzhong Road, Nanjing, 210029 People’s Republic of China; 8 Department of Integrated Traditional Chinese and Western Medicine Tumor Research Lab, Nanjing, People’s Republic of China; 9 Department of General Surgery, Yizheng Hospital, Drum Tower Hospital Group of Nanjing, 1 Huannan Road, Yizheng, 211900 People’s Republic of China

**Keywords:** Bladder cancer, *PTENP1*, Exosomes, Biomarker, Progression

## Abstract

**Background:**

Extracellular communication within the tumor microenvironment plays a critical role in tumor progression. Although exosomes can package into long non-coding RNAs (lncRNAs) to mediate extracellular communication, the role of exosomal lncRNA *PTENP1* in bladder cancer (BC) remains unclear.

**Method:**

We detected *PTENP1* expression between patients with BC and healthy controls; the expression occurred in tissues and exosomes from plasma. We assessed the diagnostic accuracy by the receiver operating characteristic curve (ROC) and the area under curve (AUC). Cell phenotypes and animal experiments were performed to determine the effect of exosomal *PTENP1*.

**Results:**

*PTENP1* was significantly reduced in BC tissues and in exosomes from plasma of patients with BC (*P* < 0.05). We found that *PTENP1* was mainly wrapped by exosomes. Exosomal *PTENP1* could distinguish patients with BC from healthy controls (AUC = 0.743; 95% confidence interval (CI) = 0.645–0.840). Normal cells secreted exosomal *PTENP1* and transmitted it to BC cells, thus inhibiting the biological malignant behavior of BC cells by increasing cell apoptosis and reducing the ability to invade and migrate (*P* < 0.05). Exosomal *PTENP1* could suppress tumor growth in vivo. Furthermore, exosomal *PTENP1* mediated the expression of PTEN by competitively binding to microRNA-17.

**Conclusion:**

Exosomal *PTENP1* is a promising novel biomarker that can be used for the clinical detection of BC. Exosomes derived from normal cells transfer *PTENP1* to BC cells, which reduce the progression of BC both in vitro and in vivo and suggest that exosomal *PTENP1* participates in normal-cell-to-bladder-cell communication during the carcinogenesis of BC.

**Electronic supplementary material:**

The online version of this article (10.1186/s12943-018-0880-3) contains supplementary material, which is available to authorized users.

## Background

Bladder cancer is one of the most prevalent malignancies of the urinary system [1, 2]. The American Cancer Society estimated that there were 79,030 new cases of bladder cancer and 16,870 deaths due to bladder cancer last year in the United States [3]. In China, the incidence and mortality rates of bladder cancer have gradually increased in recent years [4, 5]. Despite the improvement of cystoscopy, the prognosis of patients remains poor. Hence, these limitations prompted us to find new diagnostic indicators and therapeutic targets to improve the clinical efficacy of treatment for patients with bladder cancer.

Evidence has revealed that long non-coding RNAs (lncRNAs) are abnormally expressed in human cancers, including bladder cancer [6, 7]. LncRNAs are more than 200 nucleotides long with limited protein-encoding potential [8, 9], and contribute to the development of cancers by regulating several cellular processes that are crucial to tumorigenesis, such as cell proliferation, invasion, migration and apoptosis [10, 11]. Exosomes are extracellular vesicles with endocytic origin that are secreted by most cell types. They are considered as extracellular messengers between tumor cells and their microenvironment by transmitting and exchanging their diverse cargoes, which includes lncRNAs [12–15]. Additionally, exosomal lncRNAs as clinical biomarkers are stable in blood and have the capacity to distinguish whether an individual has tumors or is healthy [16, 17].

In the present study, we selected 12 lncRNAs involved in the development of multiple tumors, including *H19*, *SNHG16*, *TUG1*, *UBC1*, *UCA1*, *MALAT1*, *MEG3*, *GAS5*, *ANRIL*, *HOTAIR*, *Kcnq1ot* and *PTENP1*, and detected their expression both in tissues and exosomes of plasma. Our results revealed that *PTENP1* was significantly decreased both in bladder cancer tissues and exosomes from bladder cancer plasma. *PTENP1* is one of the pseudogene-expressed lncRNAs that plays a pivotal role in carcinogenesis [18, 19]. Nevertheless, no data are currently available regarding the biological roles of exosomal *PTENP1* in bladder cancer. The purpose of this study was to find a potential biomarker that could be used in the diagnosis of bladder cancer, and investigate if exosomal *PTENP1* intervenes in cell-cell communication, which may result in the progression of bladder cancer.

## Methods

### Study design and subjects

All subjects gave written informed consent and this study protocol was approved by the institutional review board of Nanjing Medical University. This study included analysis of plasma samples from 50 patients with bladder cancer and 60 healthy controls, as well as 20 paired tumor and adjacent normal tissues, which were obtained from patients with bladder cancer from the First Affiliated Hospital of Nanjing Medical University and Jiangsu Province Hospital of Traditional Chinese Medicine.

### Bladder cancer cell lines

Two bladder cancer cell lines (EJ and J82) and one normal human cell line (HEK 293A) were maintained under 5% CO_2_ at 37 °C in RPMI-1640 medium (Gibco BRL, Rockville, Maryland, USA) with 10% fetal bovine serum (FBS, Gibco BRL).

### Exosome isolation

The plasma and culture medium were collected and centrifuged at 3000 g for 15 min to remove cells and cellular debris. Exosomes were isolated using the Exoquick exosome precipitation solution (System Biosciences). The details of exosome isolation are shown in the Additional file 1.

### Transmission electron microscopy (TEM)

Exosomes were suspended in 100 μl of PBS and were fixed with 5% glutaraldehyde at incubation temperature and then maintained at 4 °C until TEM analysis. According to the TEM sample preparation procedure, we placed a drop of exosome sample on a carbon-coated copper grid and immersed it in 2% phosphotungstic acid solution (pH 7.0) for 30 s. The preparations were observed with a transmission electron microscope (Tecnai G2 Spirit Bio TWIN, FEI, USA).

### Western blots

Protein were prepared with a detergent buffer, and the protein concentration was determined using the bicinchoninic acid (BCA) protein assay (Beyotime Institute of Biotechnology, Shanghai, China). Equal amounts (60 μg) of protein samples were separated by a 12% gel using sodium dodecyl sulfate-polyacrylamide gel electrophoresis (SDS-PAGE) and transferred onto PVDF membranes (Millipore, Billerica, MA, USA). Monoclonal rabbit anti-TSG101 (ab125011, Abcam), monoclonal rabbit anti-CD63 (ab134045, Abcam), and anti-PTEN antibodies (#9559, Cell Signaling Technology) were incubated overnight at 4 °C with the membranes. Immune complexes were detected by enhanced chemiluminescence (Cell Signaling Technology).

### RNA isolation and quantitative real-time PCR

The total RNA was isolated from tissues and cell lines using TRIzol reagent (Invitrogen, CA, USA), and exosomal RNA was extracted from plasma and culture medium using the exoRNeasy Midi Kit (Qiagen, Valencia, CA, USA) according to the manufacturer’s protocol. The cDNA was synthesized using a high capacity cDNA reverse transcription kit (Thermo Fisher Scientific, Vilnius, Lithuania). Quantitative real-time PCR (qRT-PCR) was conducted with an ABI 7900 system (Applied Biosystems, CA, USA) and SYBR Green assays (TaKaRa Biotechnology, Dalian, China). We chose glyceraldehyde-3-phosphate dehydrogenase (GAPDH) to normalize lncRNA expression levels. The fold change in the expression of lncRNA was calculated with the formula 2^-ΔCT^. The primer sequences are shown in Additional file 1: Table S1.

### The stability of exosomal *PTENP1*

To determine the stability of exosomal *PTENP1* in plasma, we randomly selected 4 plasma samples, each of which was divided into 8 equal portions and frozen and thawed repeatedly (0 cycle, 2 cycles, 4 cycles, and 8 cycles) between − 80 °C and room temperature, or their incubation at room temperature was prolonged for 0 h, 4 h, 8 h, and 24 h. The qRT-PCR was used to detect the expression of exosomal *PTENP1*.

### Exosome labeling

Exosomes from 1.5 × 10^6^ cells were suspended in 100 μl of PBS with 1 ml of mixed PKH67 (Sigma, in Diluent C). After 4 min of incubation at room temperature, 2 ml of 0.5% bovine serum albumin (BSA) was added to terminate exosome labeling, and dyed exosomes were isolated using Exoquick exosome precipitation solution. Exosomes were suspended in 9.6 ml of basal medium, and 250 μl was added to the sub-confluent layer of EJ and J82 cells. After incubation for 3 h at 37 °C, cells were washed and fixed at room temperature. To stain the nuclei, 4′,6-diamidino-2-phenylindole (DAPI, Sigma) was added for 10 min, and the stained cells were observed with a fluorescence microscope (Zeiss, LSM700B, Germany). The details of exosome labeling are shown in the Additional file 1.

### Cell transfection

The *PTENP1* overexpression plasmid, empty vector (NC) and microRNA-17 (miRNA-17) mimics were synthesized by RiBoBio (Guangzhou, China). The lentiviral vectors containing *PTENP1*/NC were synthesized by GeneChem (Shanghai, China). Cells were transfected using Lipofectamine 2000 (Invitrogen, Carlsbad, CA, USA) transfection reagent according to the instructions.

### The malignant behaviors of bladder cancer cells

Using EJ and J82 cells, a series of assays were performed to examine the effects of *PTENP1* on malignant behaviors, including proliferation, colony formation, invasion, migration, apoptosis, and cell cycle assay. Nuclear and cytosolic fractions of 293A, EJ and J82 cells were separated using the PARIS Kit (Thermo Fisher Scientific, Vilnius, Lithuania). A detailed description of assay conditions is shown in the Additional file 1.

### Animal models

The EJ cell lines (1 × 10^7^cells in 0.1 ml of PBS) were stably transfected with *PTENP1*/ NC lentiviral vector and then injected subcutaneously into the right flank of male nude mice (5 weeks old, six mice per group). Tumor growth was examined every two days. The mice were sacrificed after two weeks, and tumor size and weight were measured. To investigate the effect of exosomal *PTENP1* in vivo, we only injected EJ cells subcutaneously into the right flank of male nude mice, and 15 days later, when tumors grew to 100 mm^3^, isolated different exosomes (10 μg) from 293A cells transfected with *PTENP1*/ NC lentiviral vector were then injected into the center of tumor every two days. After 15 days, all mice were sacrificed and tumor from different groups were measured. The animal studies were approved by the Institutional Animal Care and Use Committee of Nanjing Medical University.

### Immunohistochemistry (IHC)

Hematoxylin and eosin (H&E) staining was utilized to select representative areas. Anti-Ki67 (ab15580, Abcam) and anti-PTEN were applied for IHC. IHC staining was performed according to our previous study [20].

### Statistical analysis

Student’s *t*-test or Pearson’s χ^2^ test was used to access the differences in characteristics between bladder cancer cases and healthy controls. The threshold cycle (CT) value was analyzed using SDS 2.4 software (Applied Biosystems, Foster City, CA, USA). The receiver operating characteristic (ROC) curve was used to reflect the area under the curve (AUC) values for exosomal *PTENP1* in plasma. *P* < 0.05 was considered statistically significant. All statistical analyses were performed with SAS version 9.4 software (SAS Institute, Inc., Cary, NC, USA).

## Results

### Patient characteristics

The characteristics of the bladder cancer cases and healthy controls are summarized in Table 1. There were no differences between cases and controls in age, sex, smoking status and pack-years of smoking (*P* > 0.05). The majority of cases were in G1 (42%) and clinical grade I (82%), as well as a large proportion of cases with superficial bladder cancer (82%). The clinical characteristics of paired bladder cancer tissue samples are summarized in Additional file 1: Table S2.

**Table 1:** The characteristics of the bladder cancer cases and healthy controls

Variables	Case (*n* = 50) N (%)	Control (*n* = 60) N (%)	*P* ^a^
Age (years) (mean ± SD)	67.0 ± 9.8	66.2 ± 10.7	0.694
Sex			0.876
Male	36 (72.0)	44 (73.3)	
Female	14 (28.0)	16 (26.7)	
Smoking status			0.935
Never	38 (76.0)	46 (76.7)	
Ever	12 (24.0)	14 (23.3)	
Pack-years of smoking			0.976
0	38 (76.0)	46 (76.7)	
0–20	3 (6.0)	4 (6.7)	
> 20	9 (18.0)	10 (16.6)	
Tumor grade			
G1	21 (42.0)		
G2	14 (28.0)		
G3	15 (30.0)		
Tumor stage			
Superficial (pTa-pT1)	41 (82.0)		
Invasive (pT2-pT4)	9 (18.0)		
Clinical grade			
I	41 (82.0)		
II	6 (12.0)		
III	2 (4.0)		
IV	1 (2.0)		

^a^Student’s t-test for age between cases and controls; Two-sided χ^2^ for other variables between cases and controls

### Determination of candidate lncRNAs between bladder cancer tissues and paired normal tissues

The characteristics of 12 candidate lncRNAs are summarized in Additional file 1: Table S3. As shown in Additional file 1: Figure S1 and Table S4, the expression of *H19*, *SNHG16* and *UCA1* was significantly elevated in bladder cancer tissues, while *PTENP1* and *MEG3* were significantly decreased in bladder cancer tissues compared with adjacent normal tissues (*P* < 0.05).

### Expression of plasma exosomal *PTENP1* in patients with bladder cancer

**Fig. 1:**
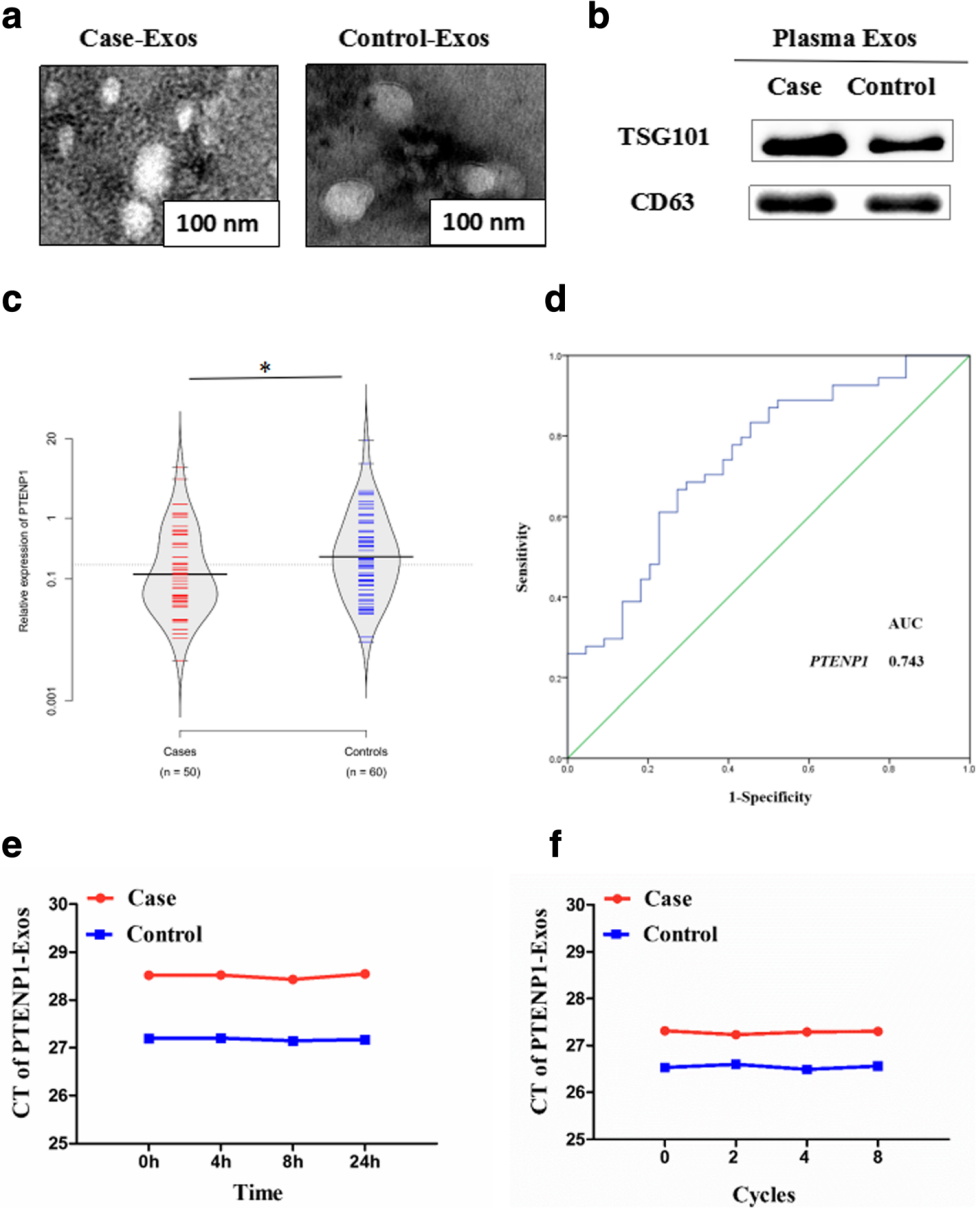
Expression of plasma exosomal *PTENP1* in patients with bladder cancer. Plasma Exosomes (Exos), exos isolated from the plasma of cases and controls. **a** Micrographs of exos isolated from the plasma of cases (left) and controls (right, bars =100 nm). **b** Western blots of TSG101 and CD63 in circulating exos. **c** qRT-PCR detection of *PTENP1* in exos from plasma. **d** ROC curves analysis of exosomal *PTENP1* signature. **e** The expression of exosomal *PTENP1* was detected after placing plasma samples at room temperature 0 h, 4 h, 8 h, and 24 h. **f** The expression of exosomal *PTENP1* was detected after freezing and thawing plasma samples repeatedly 0 cycle, 2 cycles, 4 cycles and 8 cycles. Results are presented as mean ± SD. *P < 0.05

Exosomes from plasma of patients with bladder cancer and healthy controls were isolated and characterized. TEM showed that the sizes of exosomes from cases were consistent with exosomes from healthy controls (50–120 nm, Fig. 1a). Western blots revealed the presence of the exosome markers, TSG101 and CD63 (Fig. 1b). Exosomal RNA was extracted from plasma. Subsequently, we detected the expression of *H19*, *SNHG16*, *UCA1*, *PTENP1* and *MEG3* in exosomes from plasma (Additional file 1: Figure S2A). The expression of *PTENP1* in exosomes from cases was significantly lower than that from healthy controls (*P* < 0.05, Fig. 1c). Next, we conducted a subgroup analysis by clinicopathologic features (tumor grade, tumor stage and clinical grade) and found that exosomal *PTENP1* levels gradually decreased with the deterioration of clinicopathologic features. Moreover, the expression of exosomal *PTENP1* in high clinical grade (II + III + IV) was significantly decreased compared with low clinical grade (I) (*P* < 0.05, Additional file 1: Figure S2B).

Furthermore, we evaluated the potential usefulness of exosomal *PTENP1* as a noninvasive biomarker using generated ROC curves. As shown in Fig. 1d**,** the AUC of 0.743 (95% CI = 0.645–0.840) was in exosomal *PTENP1*. The sensitivity and specificity of exosomal *PTENP1* to predict the presence of bladder cancer were 65.4% and 84.2%, respectively. Moreover, we found that exosomal *PTENP1* expression did not obviously change along with incubation time at different room temperatures (Fig. 1e), as well as at different freeze-thaw times (Fig. 1f), indicating that *PTENP1* is stable in exosomes from plasma. All these results indicated that exosomal *PTENP1* might act as a useful biomarker for discriminating patients with bladder cancer from healthy controls.

### Effect of *PTENP1* on bladder cancer cellular phenotype

**Fig. 2:**
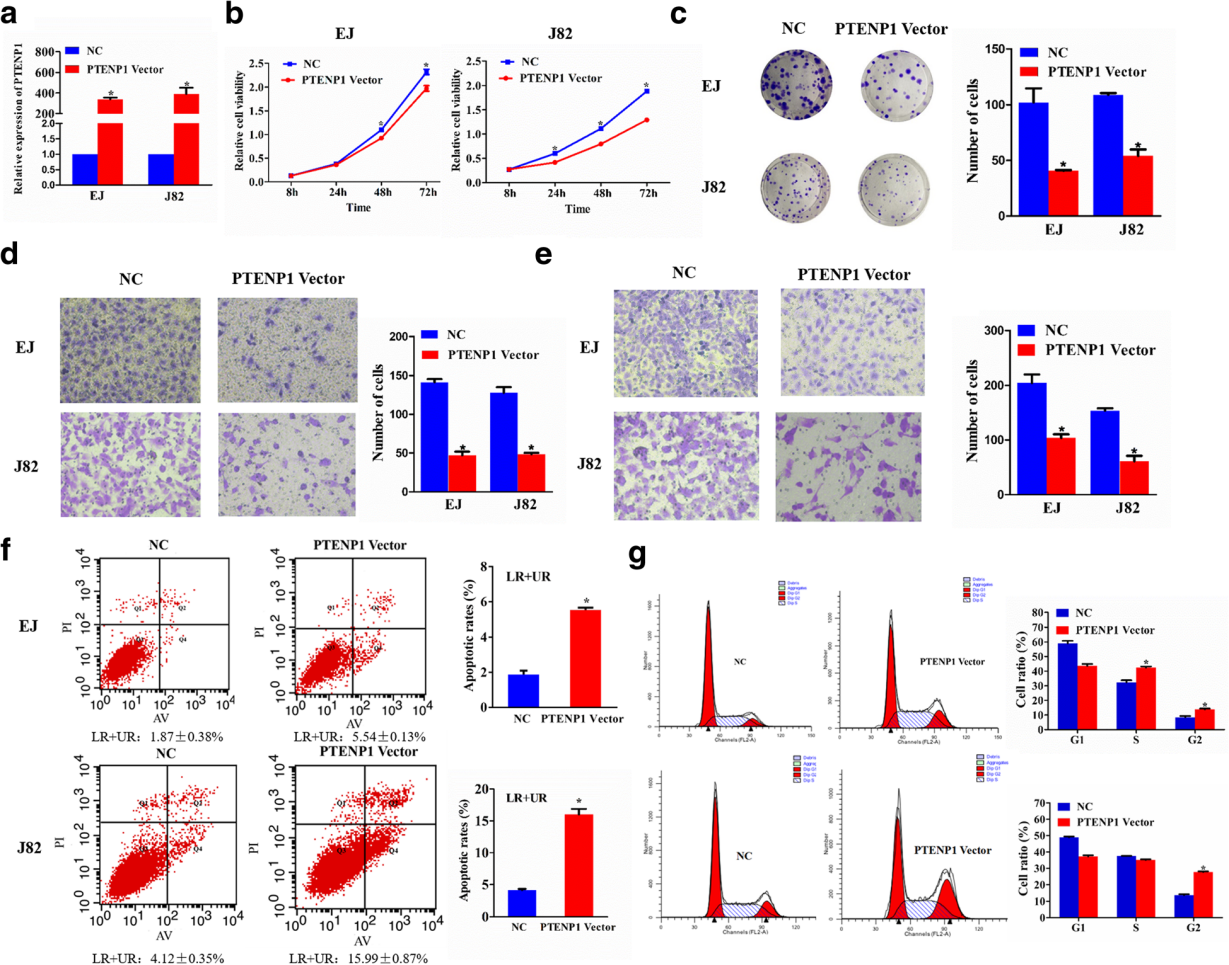
Effect of *PTENP1* on bladder cancer cellular phenotype. EJ and J82 cells were transfected with *PTENP1*-expressing plasmid or NC vector. **a** qRT-PCR detection of the *PTENP1* mRNA level. **b** A CCK8 assay detection of cell viability. **c** A colony-forming growth assay detection of cell colony formation ability. The colonies were counted and captured. **d** Representative images of invasion assays of EJ (upper) and J82 cells (lower). The number of cells were counted. **e** Representative images of migration assays of EJ (upper) and J82 cells (lower). The number of cells were counted. **f** Flow cytometry detection of the apoptosis of EJ (upper) and J82 cells (lower). **g** Flow cytometry detection of cell cycle of EJ (upper) and J82 cells (lower). Results are presented as mean ± SD. *P < 0.05. All of the experiments were performed in triplicate

In the current study, we attempted to detect the biological role of *PTENP1* in vitro, because we found that the downregulated expression of *PTENP1* in bladder cancer tissues and exosomes from bladder cancer plasma. *PTENP1*-expressing plasmid or NC vector was transfected into EJ and J82 cells. We used qRT-PCR to confirm the expression of *PTENP1* (Fig. 2a). Firstly, proliferation of EJ cells was significantly suppressed after *PTENP1* overexpression for 48 h and 72 h, whereas proliferation of J82 cells was remarkably inhibited after *PTENP1* overexpression for 24 h, 48 h and 72 h, as compared with cells transfected with NC vectors (Fig. 2b). Additionally, we obtained similar results by colony formation assays (Fig. 2c). Moreover, the two cell lines transfected with *PTENP1* overexpression for 24 h revealed remarkably impeded invasive and migratory abilities (Fig. 2d and e). Additionally, flow cytometric analysis showed that *PTENP1* overexpression induced apoptosis of EJ and J82 cells (Fig. 2f), as well as an increased percentage of EJ cells in S and G2 phase and an elevated percentage of J82 cells in G2 phase (Fig. 2g).

### Exosomal *PTENP1* serves as a mediator in intercellular communication

**Fig. 3:**
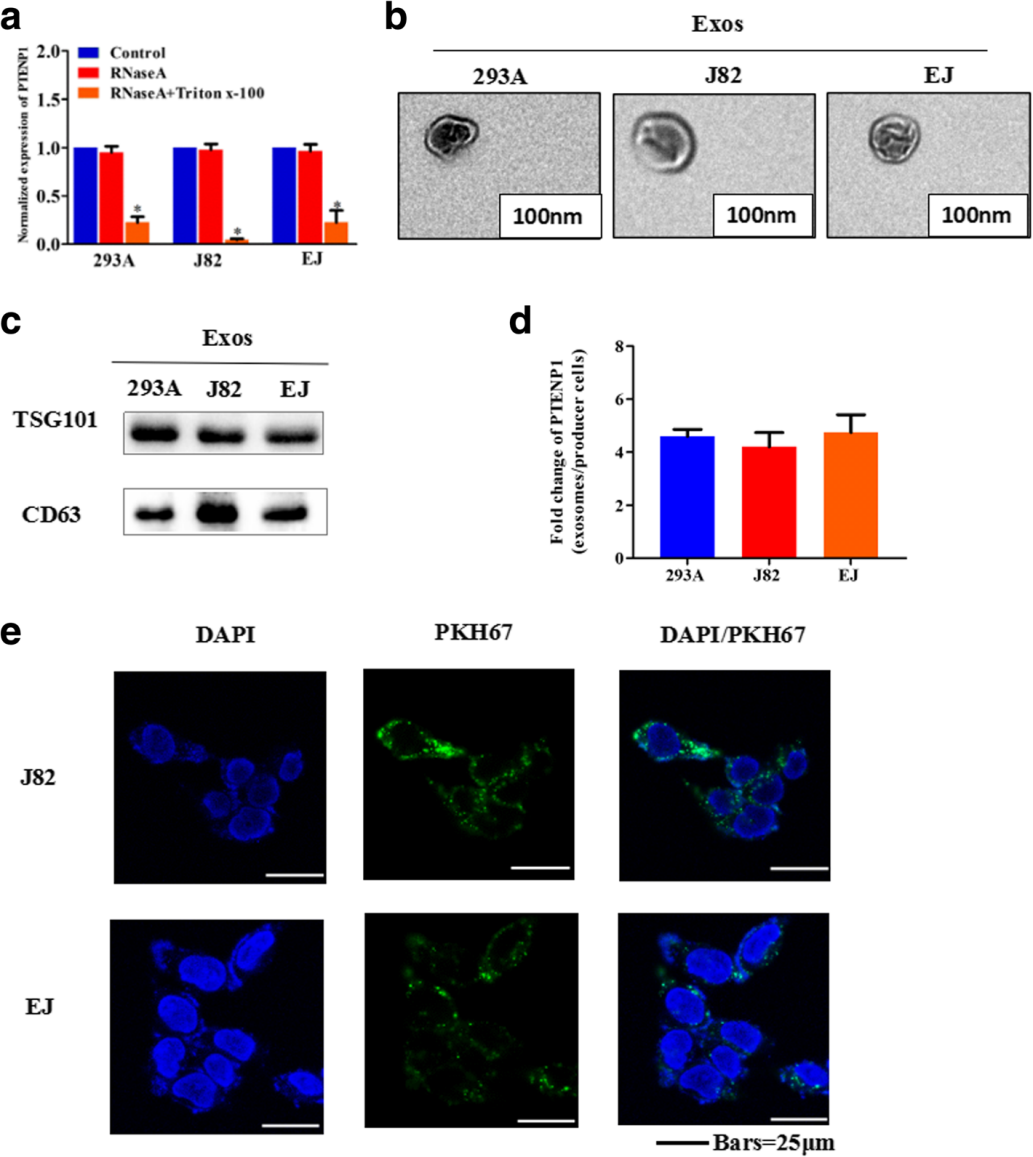
Exosomal *PTENP1* serve as a mediator in intercellular communication. Exosomes (Exos) isolated from the medium of 293A, J82 and EJ cells. **a** qRT-PCR detection of the normalized expression of *PTENP1* in the medium of 293A, J82 and EJ cells treated with RNase (2 μg/ml) alone or combined with Triton X-100 (0.1%) for 20 min. **b** Micrographs of exos isolated from 293A (left), J82 (middle) and EJ cells (right, bars =100 nm). **c** Western blots of TSG101 and CD63 in exos of cell lines. **d** qRT-PCR detection of the fold change of *PTENP1* between exos of 293A, J82 and EJ and their producer cells. **e** Exos of 293A cells were labeled with PKH67; green represents PKH67, and blue represents nuclear DNA staining by DAPI. J82 and EJ cells were incubated with exos derived from 293A cells for 3 h. Results are presented as mean ± SD. *P < 0.05. All of the experiments were performed in triplicate

We explored the existing pattern of extracellular *PTENP1*. The levels of *PTENP1* in the medium were unchanged upon RNase treatment, but were significantly reduced when simultaneously treated with RNase and Triton X-100 (Fig. 3a and Additional file 1: Figure S3A), suggesting that extracellular *PTENP1* is packaged by a membrane instead of being directly released. The sizes of exosomes were confirmed by TEM (Fig. 3b), and western blots were performed to show the presence of TSG101 and CD63 (Fig. 3c). We then found that *PTENP1* expression was significantly higher in 293A cells than in J82 and EJ cells (Additional file 1: Figure S3B). As expected, exosomal *PTENP1* levels had significantly increased in 293A cells compared with J82 and EJ cells (Additional file 1: Figure S3C). Furthermore, *PTENP1* levels were enriched by at least three-fold in exosomes compared to those in producer cells (Fig. 3d). Thus, exosomes from 293A cells contained more *PTENP1* than those from J82 and EJ cells, in agreement with our findings, and it was overexpressed in healthy controls. Then, exosomes derived from 293A cells were labeled with a green fluorescent marker, PKH67. After recipient cells (J82 and EJ cells) were incubated with labeled exosomes from 293A cells for 3 h, PKH67 was localized in the cytoplasm of recipient cells (Fig. 3e).

### Effect of exosomal *PTENP1* on bladder cancer cellular phenotype

**Fig. 4:**
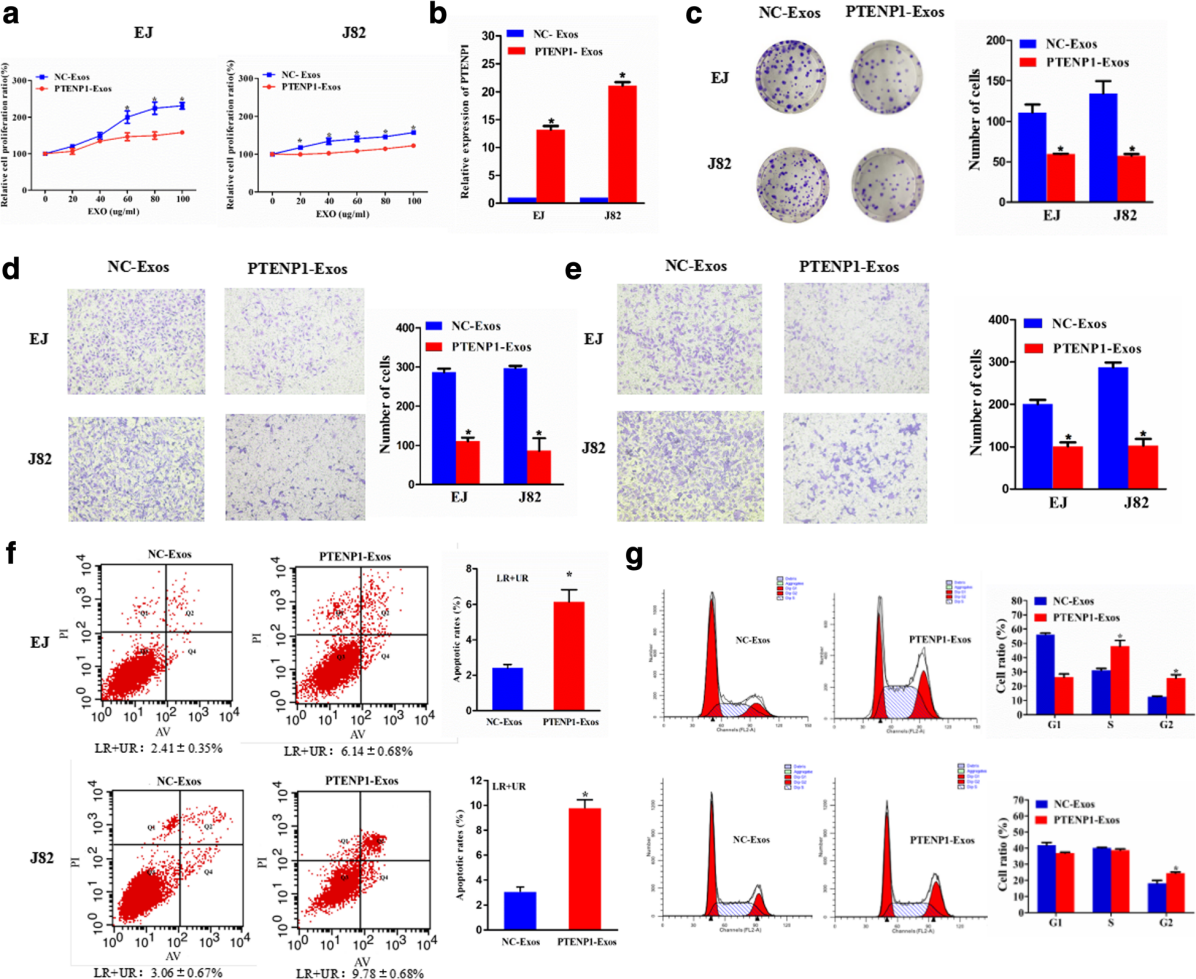
Effect of exosomal *PTENP1* on bladder cancer cellular phenotype. Exosomes (Exos) were isolated from 293A cells transfected with *PTENP1*-expressing plasmid or NC vector, namely *PTENP1*-Exos and NC-Exos, respectively. Their exosomes were extracted and added to the EJ and J82 cells for 24 h. **a** A CCK8 assay detection of cell viability. **b** qRT-PCR detection of the *PTENP1* mRNA level. **c** A colony-forming growth assay detection of cell colony formation ability. The colonies were counted and captured. **d** Representative images of invasion assays of EJ (upper) and J82 cells (lower). The number of cells were counted. **e** Representative images of migration assays of EJ (upper) and J82 cells (lower). The number of cells were counted. **f** Flow cytometry detection of the apoptosis of EJ (upper) and J82 cells (lower). **g** Flow cytometry detection of cell cycle of EJ (upper) and J82 cells (lower). Results are presented as mean ± SD. *P < 0.05. All of the experiments were performed in triplicate

Exosomes are known to play important roles in cell-cell communication, which may change the physiological function of the recipient cells by bioactive factors, including lncRNAs [21]. The frequent silencing of *PTENP1* in patients with bladder cancer and bladder cancer cell lines but not in controls indicates that *PTENP1* may act as a tumor suppressor. As described in this study, we have found that the transfer of *PTENP1* from 293A cells into EJ and J82 cells occurs via exosomes. We then predicted that exosomal *PTENP1* derived from 293A cells could change the biological function of EJ and J82 cells. However, exosomes contain diverse cargoes, such as transcriptional regulators, various RNA species, DNA and lipids [12]. To establish functions of exosomal *PTENP1*, we isolated exosomes from 293A cells transfected with *PTENP1*-expressing plasmid or NC vector, namely *PTENP1*-Exos or NC-Exos. We added *PTENP1*-Exos or NC-Exos at various concentrations (0, 20, 40, 60, 80, or 100 μg/ml) to EJ and J82 cells for 24 h. The results showed that *PTENP1*-Exos could significantly reduce proliferation of EJ and J82 cells in a concentration-dependent fashion when the concentration of exosomes was higher than 60 μg/ml (Fig. 4a). Thus, all bladder cancer cellular phenotypes were examined with the following treatment of 60 μg/ml of exosomes for 24 h in the further study. The levels of *PTENP1* in EJ and J82 cells exposed to *PTENP1*-Exos were significantly increased compared to EJ and J82 cells exposed to NC-Exos for 24 h (Fig. 4b). In addition, we used colony formation assays to obtain similar results (Fig. 4c). Furthermore, *PTENP1*-Exos inhibited the invasive and migratory abilities of EJ and J82 cells (Fig. 4d and e). Additionally, *PTENP1*-Exos increased the numbers of apoptotic EJ and J82 cells (Fig. 4f), as well as promoted cycle arrest of EJ cells at the S and G2 phase and stimulated cycle arrest of J82 cells at the G2 phase (Fig. 4g). Thus, after exposure to *PTENP1*-Exos derived from 293A cells, *PTENP1* was overexpressed and the biologically malignant behavior was inhibited in recipient EJ and J82 cells.

### Effect of *PTENP1* overexpression and exosomal *PTENP1* on tumor in vivo

**Fig. 5:**
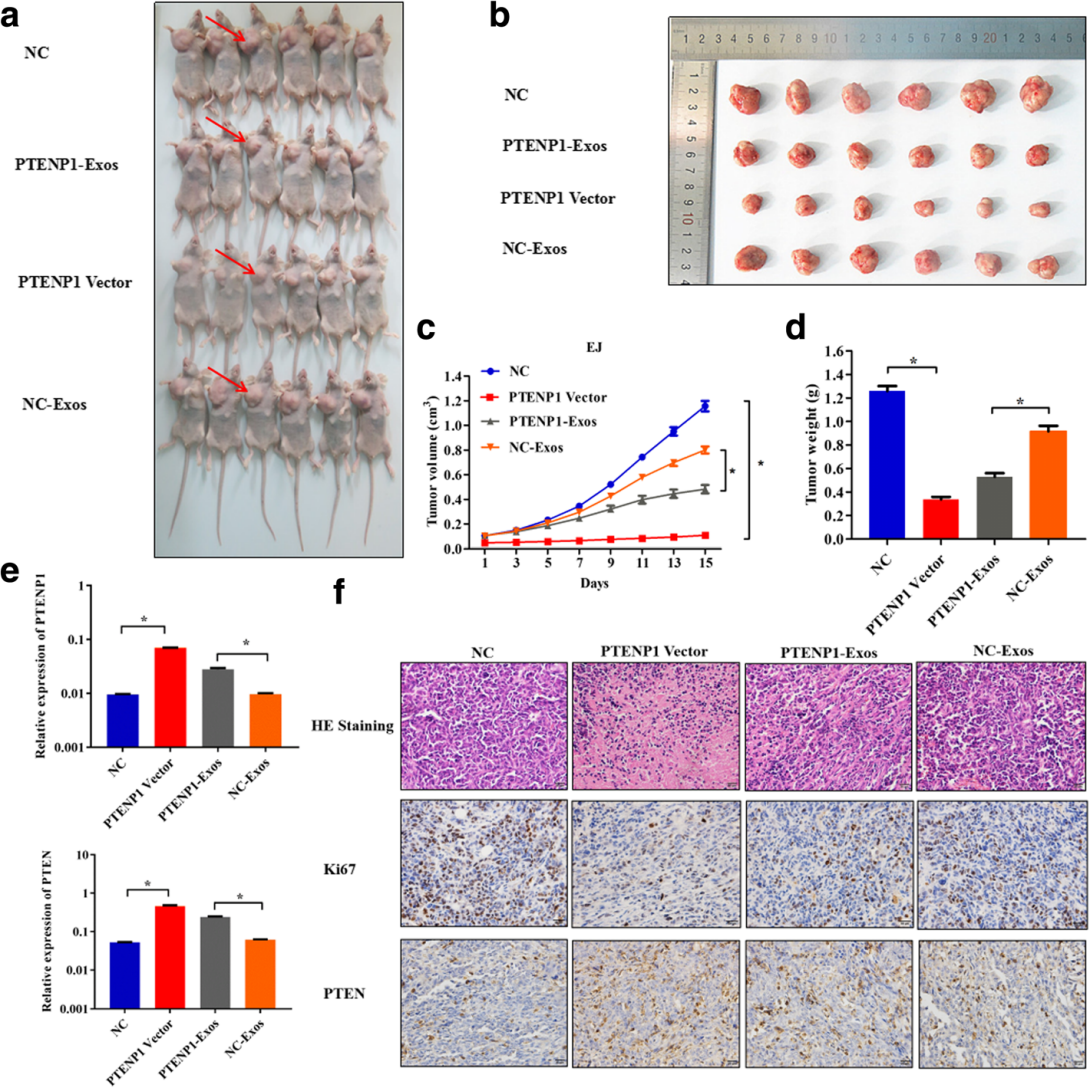
Effect of *PTENP1* overexpression and exosomal *PTENP1* on tumor in vivo. *PTENP1*/ NC lentiviral vector was transfected into EJ cells, namely *PTENP1* vector and NC, respectively. Exosomes (Exos) were isolated from 293A cells transfected with *PTENP1*/ NC lentiviral vector, namely *PTENP1*-Exos and NC-Exos, respectively. **a.** Burdened nude mice inoculated in NC, *PTENP1*-Exos, *PTENP1* vector and NC-Exos. Red arrows show position of tumor. **b.** The xenografts from nude mice inoculated in NC, *PTENP1*-Exos, *PTENP1* vector and NC-Exos. **c.** The tumor volumes were measured every two days after injection. **d.** The tumor weights in nude mice at the 15 day were determined. **e.** Detection of *PTENP1* and PTEN expression in tumor tissues of nude mice treated with NC, *PTENP1* vector, *PTENP1*-Exos and NC-Exos by qRT-PCR. **f.** H&E stained images and immunohistochemistry analysis of ki67 and PTEN expression in tumor tissues. Results are presented as mean ± SD. *P < 0.05

To further explore the role of *PTENP1* overexpression and exosomal *PTENP1* in bladder cancer in vivo, we injected EJ cells with *PTENP1*/ NC lentiviral vector or *PTENP1*/NC Exos derived from 293A cells transfected with *PTENP1*/ NC lentiviral vector into nude mice. In line with in vitro analysis, *PTENP1* overexpression significantly decreased the mean tumor weight and average tumor volume (Fig. 5a-d) as compared with NC group. As shown in Fig. 5a-d, the tumor tissues of nude mice injected with *PTENP1*-Exos also attenuated tumor size and weight. The tumor tissues of mice inoculated in *PTENP1* vector and *PTENP1*-Exos presented an increased expression of *PTENP1* (Fig. 5e). Moreover, H&E staining and immunohistochemistry for proliferation marker Ki67 was performed to investigate Ki67 expression. As shown in Fig. 5f, Ki67 expression was significantly reduced in *PTENP1* overexpression and *PTENP1*-Exos models.

### Exosomal *PTENP1* regulates PTEN expression via miR-17

**Fig. 6:**
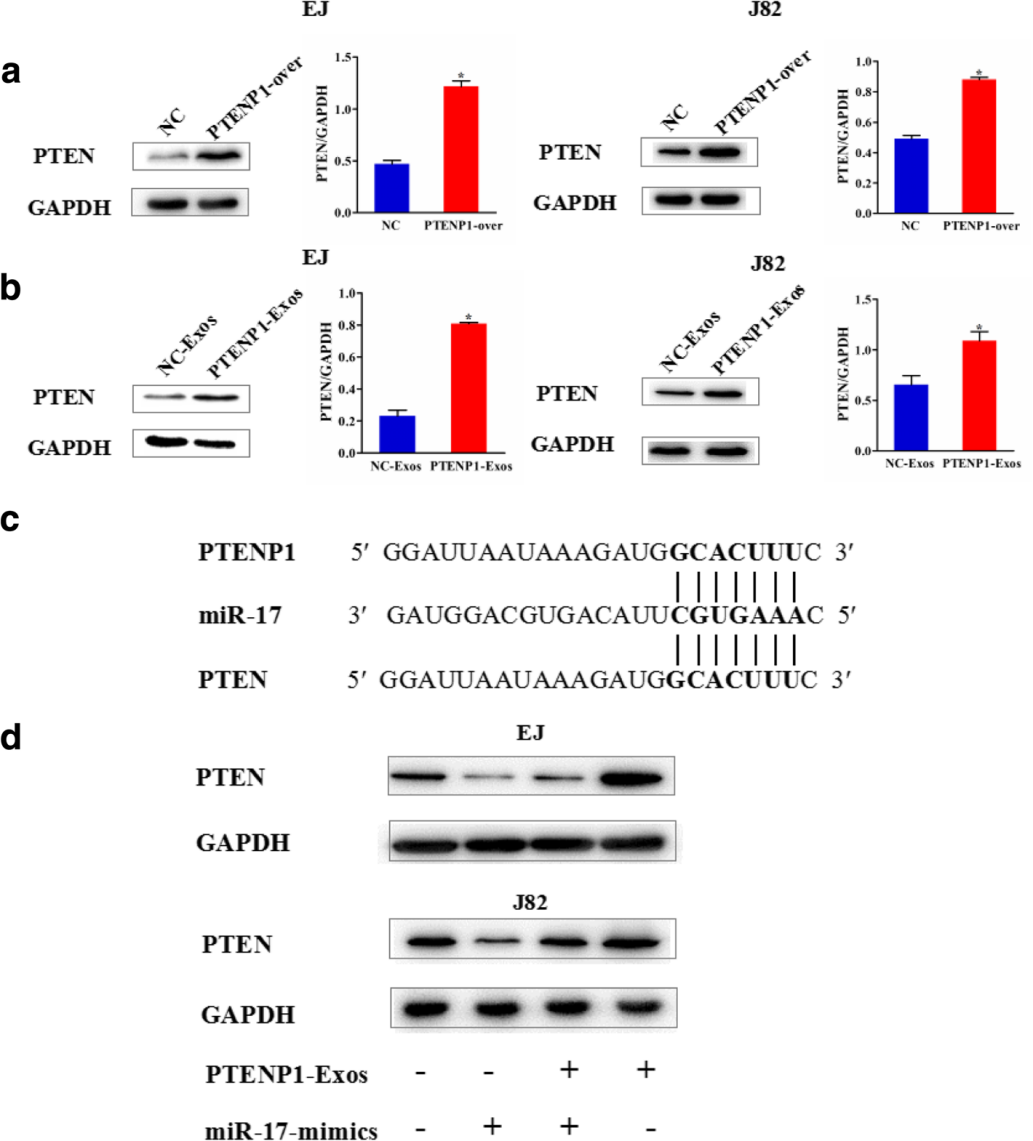
Exosomal *PTENP1* regulates PTEN expression via miR-17. **a.** Western blots of PTEN in EJ and J82 cells were transfected with *PTENP1*-expressing plasmid or NC vector. **b.** Western blots of PTEN in EJ and J82 cells were treated with *PTENP1*-Exos or NC-Exos. **c.** Putative miR-17 binding sequence in the 3′-UTR of PTEN mRNA. **d.** Western blots of PTEN in EJ and J82 cells with *PTENP1*-Exos and/ or miR-17 mimics. Results are presented as mean ± SD. *P < 0.05. All of the experiments were performed in triplicate

It is well known that *PTENP1* is a processed pseudogene of PTEN, and exerts high degree of homology to PTEN [22]. Thus, we added *PTENP1*-expressing plasmid/NC vector or *PTENP1*-Exos/NC-Exos to EJ and J82 cells, and detected PTEN expression. The results revealed that overexpression of *PTENP1* could increase PTEN expression levels (Fig. 6a). Interestingly, *PTENP1*-Exos also elevated the expression of PTEN in bladder cancer cells by western blot (Fig. 6b). Furthermore, a substantial increase of PTEN expression was identified in vivo with *PTENP1* overexpression and *PTENP1*-Exos (Fig. 5e and f). As described in this study, we have demonstrated that extracellular *PTENP1* was mainly wrapped by exosomes. These data prompted us to focus on the molecular mechanisms of exosomal *PTENP1*. Many studies have reported that *PTENP1* can competitively bind miR-17 to modulate PTEN expression [19, 23, 24]. Thus, we sought to investigate whether exosomal *PTENP1* could regulate PTEN levels by competitively binding to miR-17. The bioinformatics analysis revealed that exosomal *PTENP1* may exert its antitumor effect by regulating the expression of PTEN (Fig. 6c). As shown in Fig. 6d, miR-17 mimic reduced the expression of PTEN, whereas exosomal *PTENP1* substantially eliminated this effect.

## Discussion

**Fig. 7:**
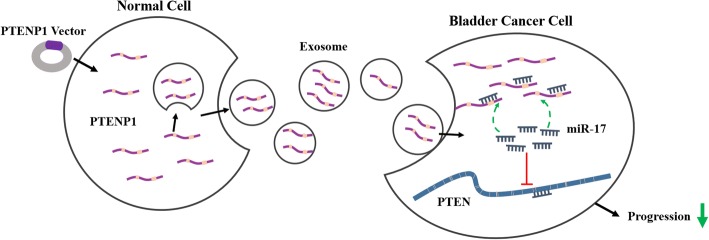
Schematic diagram of exosomal *PTENP1*-mediated bladder cancer progression. Exosomal *PTENP1* derived from normal cells transfected with *PTENP1* vector enhance *PTENP1* expression of bladder cancer cells. Exosomal *PTENP1* suppresses the progression of bladder cancer by acting as a ceRNA to competitively bind to miR-17 and regulate PTEN expression

Bladder cancer is a urinary system malignancy that is common worldwide, and its etiology is complex [2]. We identified that *PTENP1* was mainly in exosomes, and found that *PTENP1* was downregulated in bladder cancer tissues and plasma. Additionally, this study showed that normal cells could package *PTENP1* into exosomes and secrete it into bladder cancer cells, which significantly suppressed malignant behaviors of bladder cancer cells by decreasing the ability to proliferate, invade and migrate. Exosomal *PTENP1* also inhibited the growth of tumor in vivo. Thus, exosomal *PTENP1* could be a potential candidate of bladder cancer detection. In addition, we confirmed that exosomal *PTENP1* protected PTEN and suppressed biological malignant behavior of bladder cancer by sponging miR-17 (Fig. 7).

Several studies have revealed that dysregulated lncRNAs were expressed in many cancers, including bladder cancer [25, 26]. In the present study, we enrolled a total of 12 major lncRNAs with important roles in multiple tumors until December 2016, among which *Kcnq1ot1* and *PTENP1* have not been investigated in bladder cancer. This study showed that *H19*, *SNHG16*, *UCA1*, *PTENP1* and *MEG3* were aberrantly expressed in bladder cancer tissues. To date, accumulated evidences have shown that exosomal lncRNAs reflect the physiological condition of the donor cells, and being captured by recipient cells induces a series of cellular responses [27, 28]. Additionally, exosomal lncRNAs have been reported as potential cancer biomarkers for patients with bladder, gastric, colorectal and cervical cancer [28–31], suggesting that exosomal lncRNAs can be potential biomarkers for identifying cancers.

Here, we performed TEM to reveal the shapes and size of exosomes from plasma. Exosomes were also verified using the exosome markers TSG101 and CD63 [32, 33]. We then detected the expression levels of *H19*, *SNHG16*, *UCA1*, *PTENP1* and *MEG3* in exosomes from plasma. Interestingly, we found that exosomal *PTENP1* levels were downregulated in patients with bladder cancer, and lower exosomal *PTENP1* expression was associated with higher tumor and clinical grade, as well as more advanced stage. After repeated freeze-thaw cycles and incubation at room temperature, there was no significant change in the expression of exosomal *PTENP1*, suggesting that *PTENP1* is stable in the exosomes of plasma. Although our ROC analyses revealed that exosomal *PTENP1* could be useful in detecting bladder cancer, we needed to further analyze large samples to evaluate its diagnostic accuracy. Accumulating evidences have shown that lncRNAs play critical roles in controlling a variety of biochemical cellular processes, including proliferation, invasion, migration and apoptosis [34, 35]. These results led us to further examine the putative tumor suppressor function of *PTENP1* in human bladder cancer cells*.* We carried out a series of assays and found that overexpression of *PTENP1* reduced cell proliferation, migration and invasion, as well as induced cell apoptosis. Furthermore, previous studies have reported that the differentiation level of bladder cancer is assessed routinely in the clinic, poorly differentiated tumors generally exhibiting the worst prognoses [36, 37]. Therefore, we chose EJ (poorly differentiated) and J82 (well-differentiated) cell lines to evaluate the effect of *PTENP1* on cellular phenotypes. Interestingly, we found that *PTENP1* decreased the well-differentiated J82 cell viability was more obvious than poorly differentiated EJ cells in 24 h, 48 h and 72 h time points (Additional file 1: Figure S4A). Meanwhile, animal experiments also supported the tumor suppressor function of *PTENP1* in bladder cancer.

As described in our data, we have identified that *PTENP1* was mainly secreted from cells via exosomes. Exosomes are extracellular vesicles of endocytic origin and incorporate into components of donor cells, including signaling proteins, various RNA species (including lncRNA and other species), transcriptional regulators, DNA and lipids that can be taken up into neighboring or distant cell types to regulate the function of recipient cells [21, 38, 39]. The roles of exosomal lncRNAs have been intensely investigated in cancers [40, 41]. For example, exosomes released from sunitinib-resistant cells transferred *lncARSR* to sensitive cells, which induced sunitinib resistance in renal cancer [42]. Exosomal *linc-VLDL* derived from hepatocellular cancer cells exposed to sorafenib and doxorubicin was transferred into recipient tumor cells, which increased *linc-VLDL* expression and decreased chemotherapy-induced cell death in recipient cells [43]. In the present effort, we discovered that levels of *PTENP1* were increased in normal cells and their exosomes, and its expression in exosomes was approximately three times greater than that in producer cells. Similar to other reports [44, 45], fluorescence microscopy revealed that exosomes derived from normal cells that were labeled with PKH67 could transfer into bladder cancer cells. These findings indicate the possibility that 293A cells secrete exosomal *PTENP1* for transferring to the surrounding bladder cancer cells. Although normal cells exposed to exosomal lncRNAs secreted from cancer cells have been widely studied, there have been few reports describing exosomal lncRNAs released from normal cells that are transferred into cancer cells. Our results showed that *PTENP1*-Exos from 293A cells enhanced *PTENP1* expression and suppressed proliferation, migration and invasion of bladder cancer cells; in addition, it induced apoptosis in bladder cancer cells. However, exosomal *PTENP1* was not significantly reduced the viability of well-differentiated J82 cells compared to poorly differentiated EJ cells (Additional file 1: Figure S4B). This could be due to the variety of cargoes in exosomes, indicating that we will comprehensively evaluate antitumor effects of intercellular/exosomal *PTENP1* between well-differentiated J82 cells and poorly differentiated EJ cells. Consistent with our previous findings in vitro, exosomal *PTENP1* also repressed tumor growth in vivo. Hence, our findings suggest that *PTENP1* is directly transferred from normal cells to bladder cancer cells via exosomes and regulate the biological functions of bladder cancer in vitro and in vivo.

Previous studies have reported that *PTENP1* is a pseudogene-expressed lncRNA located at 9p13.3, which competitively binds miRNAs to modulate *PTEN* expression by serving as a competing endogenous RNA (ceRNA) in several cancers [46–48]. Thus, we speculated that *PTENP1* may act as a ceRNA, participating in tumorigenesis of bladder cancer. Our findings revealed that exosomal *PTENP1* could regulate PTEN levels by competing for the effect of miR-17. Our results showed that *PTENP1* and exosomal *PTENP1* were also enriched in the nucleus of cell lines, suggesting that it may also physically localizes to the *PTEN* promoter, where it attracts some chromatin modification enzymes that cause epigenetic silencing (Additional file 1: Figure S5A and B). Therefore, further studies should be performed to comprehensively investigate the biology of intercellular *PTENP1* and exosomal *PTENP1* involved in bladder cancer development and progression.

## Conclusion

In conclusion, our results indicated that exosomal *PTENP1* was a promising novel biomarker for diagnosis of bladder cancer. In addition, normal cells released exosomes containing *PTENP1,* and that exosomal *PTENP1* was transported from normal cells to bladder cancer cells, and exogenous *PTENP1* relieved the malignant phenotype of bladder cancer cells both in vitro and in vivo. Furthermore, exosomal *PTENP1* may act as a miR-17 decoy to regulate PTEN and suppress bladder cancer progression. Together, our results revealed that exosomal *PTENP1* serves as a mediator in cell-cell communication during carcinogenesis of bladder cancer.

## Additional file

Additional file 1: **Table S1.** Primer sequences in the study. **Table S2.** Clinical characteristics of paired bladder cancer tissue samples. **Table S3.** The characteristics of candidate lncRNAs until December 2016. **Table S4.** The relative expression of 12 candidate lncRNAs in paired bladder cancer tissues. **Figure S1-S5** (online). Supplementary materials (online). (DOC 2980 kb)

## Abbreviations

AUC: Area under curve
lncRNA: Long non-coding RNA
PTEN: Phosphatase and tensin homolog
*PTENP1*: Phosphatase and tensin homolog pseudogene 1
ROC: Receiver operating characteristic curve
