# Supplementary material for: Exosome–transmitted long non-coding RNA PTENP1 suppresses bladder cancer progression
Source: Mol Cancer. 2018 Oct 3;17:143. doi: 10.1186/s12943-018-0880-3 (PMC6169076; doi:10.1186/s12943-018-0880-3)
Supplement: Supplementary file 1 — Table S1. Primer sequences in the study. Table S2. Clinical characteristics of paired bladder cancer tissue samples. Table S3. The characteristics of candidate lncRNAs until December 2016. Table S4. The relative expression of 12 candidate lncRNAs in paired bladder cancer tissues. Figure S1-S5 (online). Supplementary materials (online). (DOC 2980 kb) [file 12943_2018_880_MOESM1_ESM.docx]

**Supplemental Materials**

**Supplemental Data**

**Table S1. Primer sequences in the study**

| H19 | Forward (5’-3’) | GCACCTTGGACATCTGGAGT |
| --- | --- | --- |
|  | Reverse (5’-3’) | TTCTTTCCAGCCCTAGCTCA |
| SNHG16 | Forward (5’-3’) | ACAGGATGCCGTCTTGTGTT |
|  | Reverse (5’-3’) | GGTGGCAACCCATTAGCAGA |
| TUG1 | Forward (5’-3’) | CTGAAGAAAGGCAACATC |
|  | Reverse (5’-3’) | GTAGGCTACTACAGGATTTG |
| UBC1 | Forward (5’-3’) | CCTGCTTGGAAACTAATGACC |
|  | Reverse (5’-3’) | AGGCTCAACTTCCCAGACTCA |
| UCA1 | Forward (5’-3’) | TTAAGGTGTCCACGCAGTCC |
|  | Reverse (5’-3’) | TGGGGATTACTGGGGTAGGG |
| MALAT1 | Forward (5’-3’) | GTGAGCAAACTGTGTTGGCGTG |
|  | Reverse (5’-3’) | CATCGAGGTGAGGGGTGAAGGG |
| MEG3 | Forward (5’-3’) | GGGCATTAAGCCCTGACCTT |
|  | Reverse (5’-3’) | CCTTGGGGAGGGAAACACTC |
| GAS5 | Forward (5’-3’) | CACUCUGAGUGGGACAAGCUCUUCA |
|  | Reverse (5’-3’) | UGAAGAGCUUGUCCCACUCAGAGUG |
| ANRIL | Forward (5’-3’) | TGCTCTATCCGCCAATCAGG |
|  | Reverse (5’-3’) | GGGCCTCAGTGGCACATACC |
| HOTAIR | Forward (5’-3’) | GGTAGAAAAAGCAACCACGAAGC |
|  | Reverse (5’-3’) | ACATAAACCTCTGTCTGTGAGTGCC |
| Kcnq1ot1 | Forward (5’-3’) | CCAGCATAGCCTTATGGA |
|  | Reverse (5’-3’) | GCTGTCCGGGCCTCTGATCTCCGAT |
| PTENP1 | Forward (5’-3’) | TCTCTCATCTCCCTCGCCTGA |
|  | Reverse (5’-3’) | AGCCGTGATGGAAGTTTGAATG |
| PTEN | Forward (5’-3’) | AATGTTCAGTGGCGGAACTTGC |
|  | Reverse (5’-3’) | AACTTGTCTTCCCGTCGTGTGG |
| GAPDH | Forward (5’-3’) | CCGGGAAACTGTGGCGTGATGG |
|  | Reverse (5’-3’) | AGGTGGAGGAGTGGGTGTCGCTGTT |
| U6 | Forward (5’-3’) | CTCGCTTCGGCAGCACA |
|  | Reverse (5’-3’) | AACGCTTCACGAATTTGCGT |

**Table S2. Clinical characteristics of paired bladder cancer tissue samples**

| **No.** | **Age (years)** | **Sex** | **Smoking status** | **Tumor grade** | **Tumor stage** |
| --- | --- | --- | --- | --- | --- |
| 1 | 86 | Male | No | High grade | Non-muscle invasive |
| 2 | 67 | Female | No | Low grade | Invasive |
| 3 | 68 | Male | No | High grade | Non-muscle invasive |
| 4 | 81 | Female | No | Low grade | Non-muscle invasive |
| 5 | 72 | Male | Yes | High grade | Non-muscle invasive |
| 6 | 55 | Female | No | Low grade | Non-muscle invasive |
| 7 | 67 | Male | No | Low grade | Non-muscle invasive |
| 8 | 73 | Male | Yes | High grade | Non-muscle invasive |
| 9 | 80 | Male | No | Low grade | Non-muscle invasive |
| 10 | 76 | Male | Yes | High grade | Non-muscle invasive |
| 11 | 79 | Female | No | Low grade | Invasive |
| 12 | 63 | Male | Yes | High grade | Non-muscle invasive |
| 13 | 86 | Male | No | High grade | Non-muscle invasive |
| 14 | 79 | Male | No | High grade | Non-muscle invasive |
| 15 | 71 | Male | Yes | High grade | Non-muscle invasive |
| 16 | 79 | Female | No | Low grade | Invasive |
| 17 | 79 | Male | No | High grade | Non-muscle invasive |
| 18 | 62 | Female | No | Low grade | Invasive |
| 19 | 72 | Male | No | High grade | Non-muscle invasive |
| 20 | 48 | Male | No | High grade | Non-muscle invasive |

**Table S3. The characteristics of candidate lncRNAs until December 2016**

| **IncRNA** | **Dysregulation** | **Location** | **Cancer** | **Bladder Cancer** |
| --- | --- | --- | --- | --- |
| H19 | UP | 11:2016406..  2019065 | bladder, gastric, cervical | Reported |
| SNHG16 | UP | 17:74553846..  74561430 | bladder, colorectal | Reported |
| TUG1 | UP | 22: 31365634..  31375381 | bladder, breast, pancreas, endometrial | Reported |
| UBC1 | UP | 4: 39699664..  39784412 | bladder, colorectal, gastric | Reported |
| UCA1 | UP | 19:15939757..  15947131 | bladder, tongue squamous cell carcinomas | Reported |
| MALAT1 | UP | 11: 65265224..  65273940 | bladder, ovarian, breast,  thyroid, gastric,  esophageal | Reported |
| MEG3 | Down | 14:101292445..  101327363 | bladder, lung | Reported |
| GAS5 | Down | 1: 173833039..  173837125 | bladder, prostate, thyroid | Reported |
| ANRIL | UP | 9: 21994790..  22121096 | bladder, lung, ovarian, hepatocellular carcinoma | Reported |
| HOTAIR | UP | 12: 54356092..  54368740 | bladder, ovarian, gastric,  colorectal, glioma, lung,  cervical, urothelial, breast, carcinoma, pancreas | Reported |
| Kcnq1ot1 | UP | 11: 2629558..  2721228 | Nephroblastoma, hepatoblastoma | Not Reported |
| PTENP1 | Down | 9:33673502..  33677418 | prostate,  endometrial melanoma | Not Reported |

**Table S4. The** **relative expression of 12 candidate lncRNAs in paired bladder cancer tissues**

| **lncRNA** | **Expression level (mean ± SD)** | | ***P*^a^** |
| --- | --- | --- | --- |
|  | **Cancer** | **Normal** |  |
| H19 | 0.34±0.62 | 0.06±0.09 | 0.050 |
| SNHG16 | 0.13±0.15 | 0.05±0.04 | 0.048 |
| UCA1 | 0.44±0.70 | 0.12±0.11 | 0.032 |
| PTENP1 | 0.03±0.03 | 0.05±0.03 | 0.015 |
| MEG3 | 0.03±0.08 | 0.18±0.31 | 0.037 |
| MALAT1 | 5.81±8.85 | 2.94±3.09 | 0.179 |
| ANRIL | 0.02±0.02 | 0.03±0.02 | 0.087 |
| TUG1 | 0.04±0.04 | 0.04±0.04 | 0.963 |
| GAS5 | 0.34±0.50 | 0.41±0.58 | 0.682 |
| HOTAIR | 0.05±0.08 | 0.07±0.07 | 0.222 |
| Kcnq1ot1 | 0.01±0.01 | 0.02±0.01 | 0.166 |
| UBC1 | 0.07±0.14 | 0.08±0.10 | 0.906 |

^a^ Student’s t-test for relative expression of lncRNA between cancer and normal tissues.

**Figure Legend**

**Figure S1. Determination of candidate lncRNAs between bladder cancer tissues and paired normal tissues.** qRT-PCR detection of the relative expression of 12 candidate lncRNAs in paired BC tissues (n=20). The data are presented as a fold-change in the tumor tissue relative to the normal tissue. Results are presented as mean ± SD. **P* < 0.05.

**Figure S2, related to Figure 1. Expression of plasma exosomal *PTENP1* in patients with bladder cancer.** Plasma Exosomes (Exos), exos isolated from the plasma of cases and controls. **A.** qRT-PCR detection of the relative expression of 5 candidate lncRNAs in plasma exos. **B.** qRT-PCR detection of the relative expression of *PTENP1* in patients of different tumor grade, tumor stage and clinical grade. Results are presented as mean ± SD. **P* < 0.05.

**Figure S3, related to Figure 3.** **Exosomal *PTENP1* serve as a mediator in intercellular communication.** Exosomes (Exos) isolated from the medium of 293A, J82 and EJ cells, namely 293A exos, J82 exos and EJ exos, respectively. **A.** qRT-PCR detection of the relative expression of *PTENP1* in the medium of 293A, J82 and EJ cells treated with RNase (2 μg/ml) alone or combined with Triton X-100 (0.1%) for 20 min. **B.** qRT-PCR detection of the relative expression of *PTENP1* in cell lines. **C.** qRT-PCR detection of the relative expression of *PTENP1* in 293A exos, J82 exos and EJ exos. Results are presented as mean ± SD. **P* < 0.05. All of the experiments were performed in triplicate.

**Figure S4, related to Figure 2 and Figure 4. Effect of *PTENP1/* exosomal *PTENP1* on bladder cancer cellular phenotype.** Totally 4.0 × 10^3^ EJ and J82 cells were transfected with *PTENP1*-expressing plasmid or NC vector, namely *PTENP1* vector and NC, respectively. Exosomes (Exos) were isolated from 293A cells transfected with *PTENP1*-expressing plasmid or NC vector, namely *PTENP1*-Exos or NC-Exos, respectively. The 60 μg/ml of *PTENP1*-Exos and NC-Exos were extracted and added to the EJ and J82 cells for 24h. The viability ratios of NC and NC-Exos were used to establish the 100% level. **A.** CCk8 detection of the cell viability in EJ and J82 cells between *PTENP1* vector and their corresponding NC, related to Figure 2B. **B.** CCk8 detection of the cell viability in EJ and J82 cells between *PTENP1*-Exos and their corresponding NC-Exos, related to Figure 4A. Results are presented as mean ± SD. **P* < 0.05. All of the experiments were performed in triplicate.

**Figure S5, related to Figure 6. Exosomal *PTENP1* regulates *PTEN* expression via miR-17.** *U6* and *GAPDH* were used as a nucleus marker or cytoplasm marker, respectively. **A.** qRT-PCR detection of the expression *PTENP1* in nucleus or cytoplasm of EJ and J82 cells were treated with *PTENP1*-Exos. **B.** qRT-PCR detection of the expression *PTENP1* in cell nucleus or cytoplasm. Results are presented as mean ± SD. All of the experiments were performed in triplicate.

**Figure S1.**

**
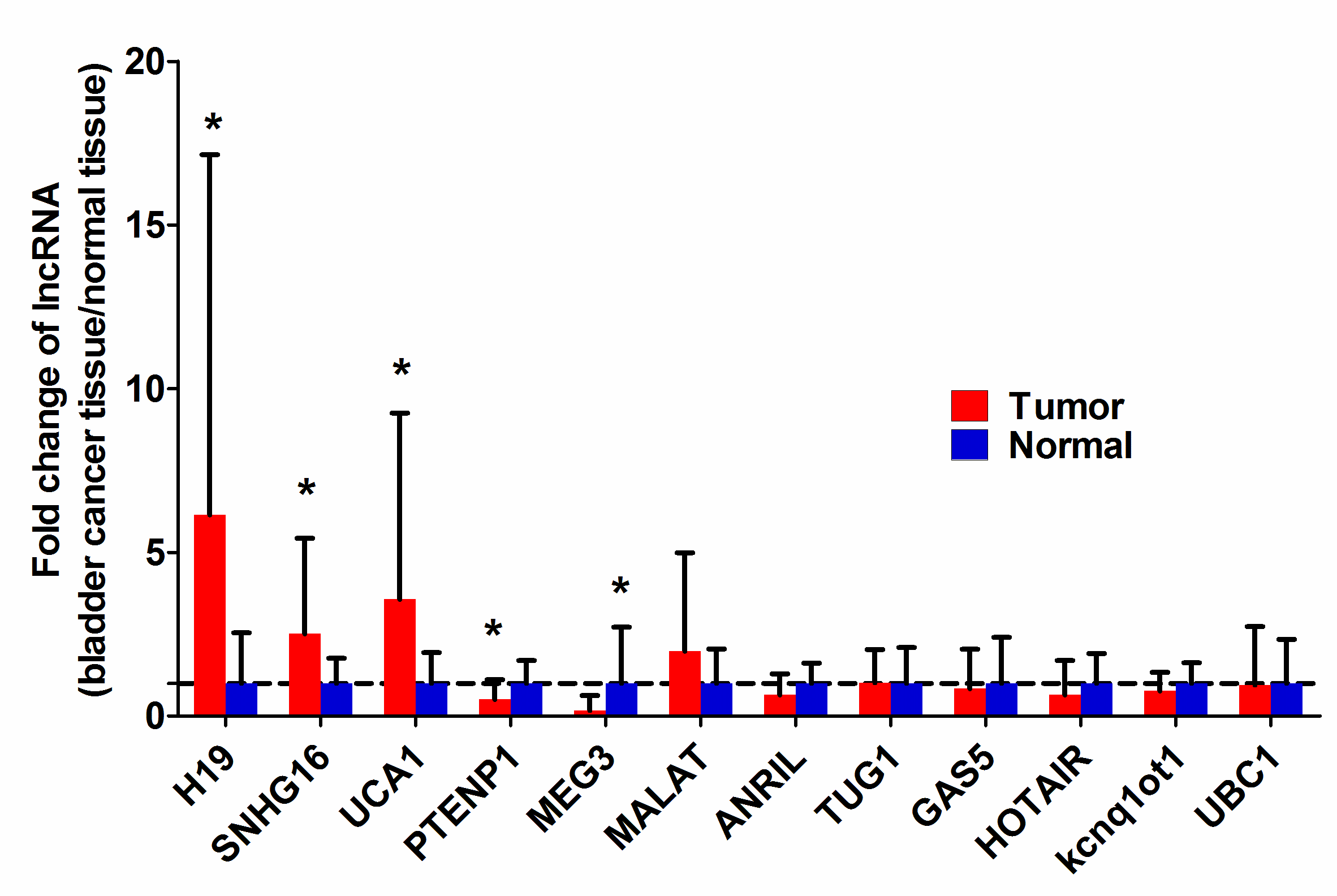
**

**Figure S2.**


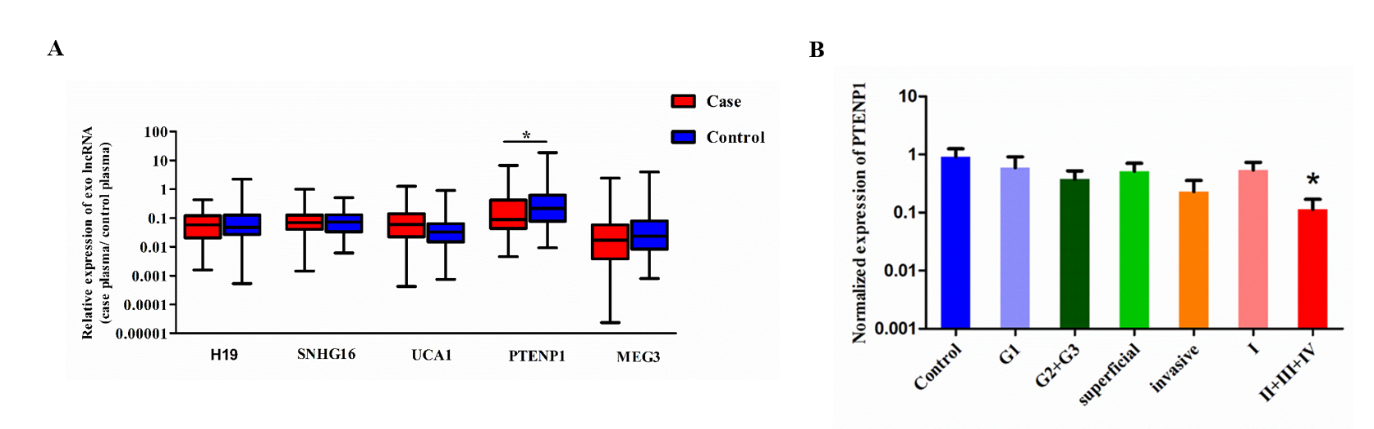


**Figure S3.**

**
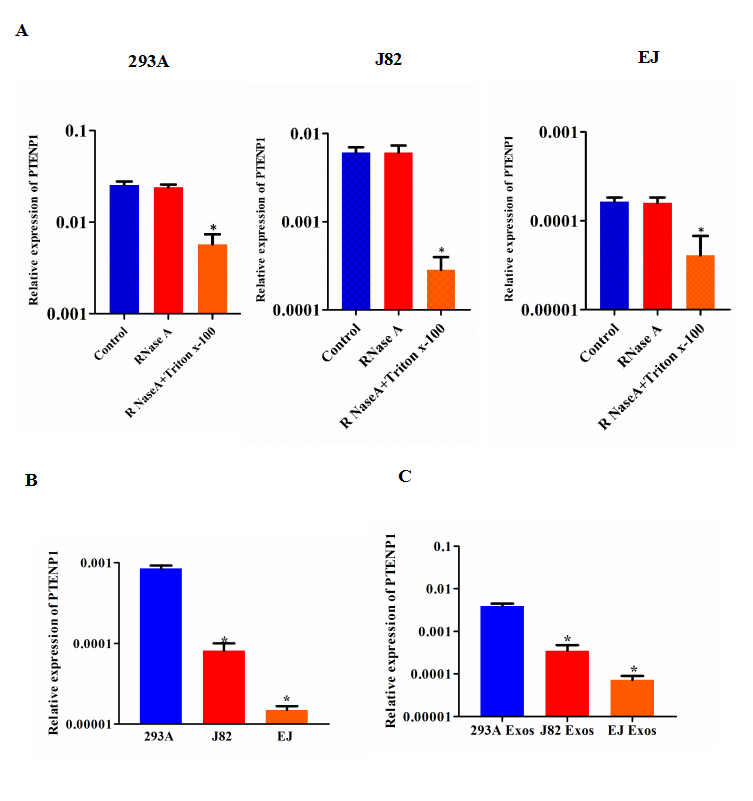
**

**Figure S4.**

**
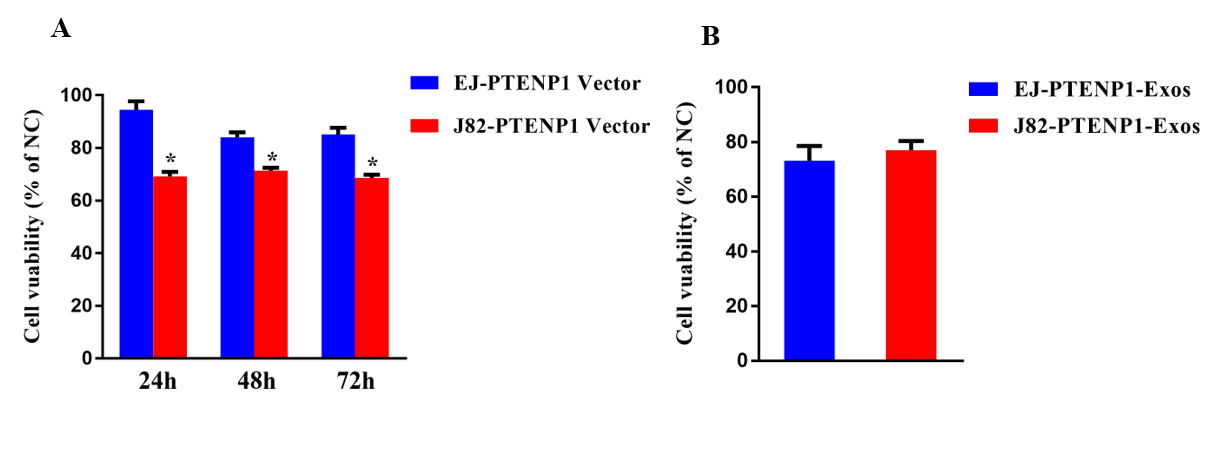
**

**Figure S5.**


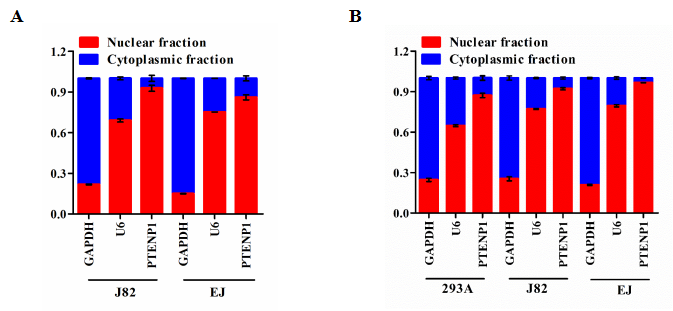


**Supplemental experimental procedures**

**Exosome isolation**

The plasma and culture medium were collected and centrifuged at 3000 g for 15 min to remove cells and cellular debris. Then, we filtered the supernatant through a 0.22- μm PVDF filter (Millipore). In the case of filtered plasma, an appropriate volume of Thrombin was added to the plasma and centrifuged at 10,000 rpm, 5 minutes to make them compatible with ExoQuick exosome precipitation. Then, we added the appropriate volume of ExoQuick exosome precipitation solution (System Biosciences) to the Thrombin-treated plasma and filtered culture medium. After refrigeration for 24 h, the ExoQuick/biofluid mixture was centrifuged at 1500 g for 30 min, and the supernatant was removed. The exosomes appear as a beige or white pellet at the bottom of the vessel.

**Exosome labeling**

Exosomes from 1.5 × 10^6^ cells were suspended in 100 μl of PBS with 1 ml of Diluent C. Then, the 4ul of PKH67 (Sigma, in Diluent C) and 1ml of Diluent C were mixed and added to the exosomes. After 4 min of incubation at room temperature, 2 ml of 0.5% bovine serum albumin (BSA) was added to terminate exosomes labeling, and dyed exosomes were isolated by using Exoquick exosome precipitation solution. Exosomes were suspended in 9.6 ml of basal medium, and 250 μl was added to sub-confluent layer of EJ and J82 cells. After incubation for 3 h at 37 °C, cells were washed twice with PBS, and incubated with 1 ml of 4 % paraformaldehyde for 30 min at room temperature. Then, cells were washed three times with PBST (3.5 g of Na_2_HPO_4_.12H_2_O, 0.25 g of NaH_2_PO_4_.2H_2_O, 4.5 g of NaCl, and 0.25 ml of TWEEN dissolved in 500 ml of double distilled water). To stain the nuclei, the 4’,6-diamidino-2-phenylindole (DAPI, Sigma) was added for 10 min, and stained cells were observed on a fluorescence microscope (Zeiss, LSM700B, Germany).

**Cell proliferation assay**

Approximately 4.0 × 10^3^ EJ and J82 cells that were transfected with *PTENP1* overexpression/NC vectors or incubated with exosomes were plated in 96-well plates. Cell proliferation was performed by using the Cell Counting Kit-8 (Dojindo Laboratories, Kumamoto, Japan) according to manufacturer’s protocol. The absorbance was evaluated at 450 nm by the Infinite M200 spectrophotometer (Tecan, Switzerland). We performed all experiments in triplicate.

**Colony formation assays**

A total of 24 h after transfection or exosomes incubation, about 200 cells were placed onto the 6-well plate and maintained in RPMI-1640 with 10% FBS, replacing the medium every 4 days. After 2 weeks, the colonies were fixed using 95% methanol and stained with 0.1% crystal violet. We performed all experiments in triplicate.

**Cell invasion assay**

To investigate the effect of *PTENP1* and exosomal *PTENP1* on EJ or J82 cell invasion. The day before cells seeded on the upper chamber of the trans-well (8-μm pore size, Corning, MA, USA), Matrigel (Becton Dickinson Labware, Bedford, MA) was added to the upper chamber. Twenty-four hours after transfection or exosomes incubation, a total of 4.0 × 10^4^ EJ or 5.0× 10^4^ J82 cells suspended in 100 μl of serum-free medium were seeded into the upper chamber of the trans-well. RPMI-1640 with 10% FBS was added to the lower chamber. After 24 h of culture at 37˚C, the cells of upper chamber were removed, and the cells on the lower chamber were fixed in 95% methanol for 30 min and stained with 0.1% crystal violet for 20 min. The stained cells were counted at five fields per membrane under microscopic inspection. We performed all experiments in triplicate.

**Cell migration assay**

Similar to invasion assay, migration assay was also performed by trans-well filters.

About 2.0 × 10^4^ EJ or 3.0× 10^4^ J82 cells were suspended in 100 μl of serum-free medium and seed into upper chamber. The following process is consistent with cell invasion assay. We performed all experiments in triplicate.

**Flow-cytometric analysis for apoptosis and cell cycle**

For apoptosis assay, the *PTENP1* overexpression/NC vectors or exosomes was cultured in 6-well plate for 24 h, and then stained with Annexin V (FITC-conjugated) (BD Biosciences, Erembodegem, Belgium) and 7-amino-actinomycin (7-AAD; BD Biosciences). Apoptosis assay was evaluated by flow cytometry. For cell cycle assay, cells were collected and fixed using 70% ethanol at -20 ˚C for 18 h, and stained with 500 ul of propidium iodide (PI) for 15 min.
